# Anomalous Gold Concentrations in Hypersaline Wetland Sediments (Laguna Honda, South Spain) Caused by Nanoparticles Used in Agricultural Practices: Environmental Transformation

**DOI:** 10.3390/toxics12030223

**Published:** 2024-03-18

**Authors:** Antonio Medina-Ruiz, Juan Jiménez-Millán, Isabel Abad, Rosario Jiménez-Espinosa

**Affiliations:** Departmento de Geología and Centro de Estudios Avanzados en Ciencias de la Tierra, Energía y Medio Ambiento (CEACTEMA), Universidad de Jaén, Campus Las Lagunillas, 23071 Jaén, Spain; medina@ujaen.es (A.M.-R.); miabad@ujaen.es (I.A.); respino@ujaen.es (R.J.-E.)

**Keywords:** gold, nanoparticles, hypersaline wetland, aggregation, dissolution, bacterial influence, Laguna Honda

## Abstract

Illite-rich sediments from the Laguna Honda wetland, an eutrophicated hypersaline wetland with waters enriched in Mg and Ca surrounded by olive groves in the Guadalquivir Basin River (South Spain), are polluted by elevated concentrations of gold (up to 21.9 ppm) due to agricultural practices. The highest gold contents appear in the shore sediments of the lake, where up to 20 µm homoaggregates of fused gold nanoparticles (AuNp) are found. Small nanoaggregates of up to six fused gold nanoparticles and very few isolated nanoparticles around 1 nm in size can also be observed to form heteroaggregates of AuNp-mica, especially in the deeper sediments in the central part of the wetland, where Au concentrations are lower (up to 1.89 ppm). The high nanoparticle concentration caused by the inappropriate application of pesticides favors nanoparticle collision in the wetland’s Mg- and Ca-rich waters and the fast coagulation and deposition of Au homoaggregates in the gold-rich shore sediment of the lake. The interaction of gold nanoparticles with the abundant illite particles in the wetland’s hypersaline waters promotes the simultaneous formation of low-density Au-illite heteroaggregates, which are transported and deposited in the less-rich-in-gold sediments of the central part of the lake. The small sizes of the isolated AuNp and AuNp-fused contacts of the aggregates suggest modifications in the original nanoparticles involving dissolution processes. The presence of bacterial communities resistant to heavy metal stress (*Luteolibacter* and *Maricaulis*), as well as the activity of sulfate-reducing bacteria (SRB) and particularly sulfur-oxidizing bacteria (SOB) communities from the shore sediments, favored the high-Eh and low-pH conditions adequate for the destabilization and transport of AuNp.

## 1. Introduction

The use of gold nanoparticles (AuNp) has been quickly and expansively incorporated into agricultural practices due to its capability to enhance the growth of plants and increase crop yield. The application of AuNp is commonly customized to transport or transfer specific compounds that are accurately delivered to produce accurate fertilization processes and the supply of specific micronutrients or particular pesticide treatments, e.g., [1,2,3,4,5,6].

The extended use of these substances is increasing their environmental exposure; therefore, efforts to understand and report the environmental fate of AuNp are required. The environmental stability of AuNp has been considered very high, but their accumulation and transformations are controlled by environmental variables (e.g., pH, Eh, salinity, aqueous ionic strength, organic matter content, and presence of humic acids, among others) and nanoparticle properties [5,7], which means that some environmental compartments, such as sediments deposited in areas with high agricultural pressure, can become major sinks for these nanoparticles. The environmental state of nanomaterials in aquatic systems is defined by the interplay between the growth, dissolution, and aggregation of nanoparticles [8]. Surette and Nason [9] highlighted the importance of the aggregation of similar (homoaggregation) and dissimilar (heteroaggregation) nanoparticles in the transport and deposition of engineered nanoparticles in aquatic media. When the interactions between heavy metal nanoparticles produce heavier and bigger homoaggregates, the deposition of particles is promoted relative to their transport [10] and nanoparticles end up in sediments near the nanoparticles’ release points. In contrast, if nanoparticles do not aggregate or heteroaggregation with lighter particles occurs, they can persist as mobile particles and be transported far away from the emission points [9,11]. Moreover, although AuNps have been thought to be rather stable to dissolution in many environmental conditions, some experimental studies have revealed that some chemical substances produced by microbial and plant metabolism (such as cyanide or thiosulfate) can promote the oxidation of Au^0^ nanoparticles and the formation of complexes resulting in Au dissolution in soils, sediments, and aquatic systems [12,13].

Mediterranean olive culture is seriously challenged by the effects of climate change, which has motivated an increase in the use of agrochemicals in order to maintain productivity, resulting in potential environmental hazards [14]. The interest in metal nanoparticles in this sector as a sustainable alternative has grown due to their ability to slowly release nutrients and pesticides in a controlled manner [14,15,16]. Spain is the biggest olive oil manufacturer worldwide [17], and around 1.6 million ha of land in Andalusia (South of Spain) is dedicated to olive culture, especially in the Guadalquivir River Basin of the Jaén and Cordoba provinces [18]. This area is characterized by the presence of frequently eutrophicated wetlands completely surrounded by olive groves, which are usually considered hotspots for controlling the biogeochemical transformations of nutrients (P and N coming from agricultural practices) [19] and can be sensitive systems for the concentration and modification of pesticides that may contain nanoparticles used in olive culture.

Therefore, understanding the transformations that gold nanoparticles undergo in complex environmental systems is a longstanding research problem for predicting the fate of metal nanoparticles in these systems. This study focuses on the modification, concentration, and fate of AuNp, possibly emitted from agrochemicals, in the sediments of a hypersaline wetland enriched with organic matter surrounded by olive groves in the Guadalquivir Basin River (South Spain). We report the images of the biggest AuNp aggregates obtained by means of electron microscopy in sediments from a protected wetland. The main objective of this study is to contribute to research related to the knowledge of factors controlling the transformation of gold nanoparticles in hypersaline detrital sediments, revealing (a) the role of the detrital inputs (micas) in the transport and deposition of the gold nanoparticles via the textural development of different types of aggregates and (b) the influence of the physicochemical conditions of water and sediments controlled by the presence of organic matter and microorganism activity on the hazards for Au mobilization.

## 2. Materials and Methods

### 2.1. Study Area and Materials

**Figure 1:**
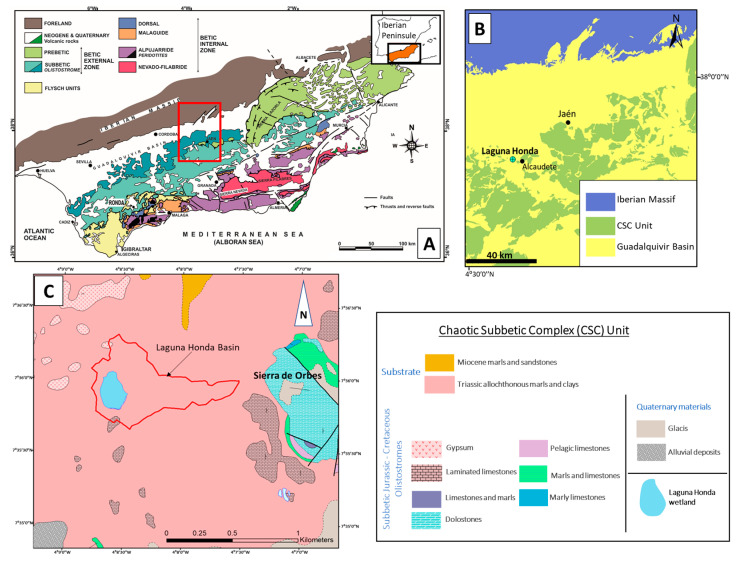
Location of the study area. (**A**) Geographical setting and global geological context of the Betic Cordillera and the study area (modified from Figure 3 in Sanz de Galdeano [23]). (**B**) Geological map of the chaotic subbetic complexes (CSCs) near the study area. (**C**) Local geological map of the Laguna Honda area.

**Figure 2:**
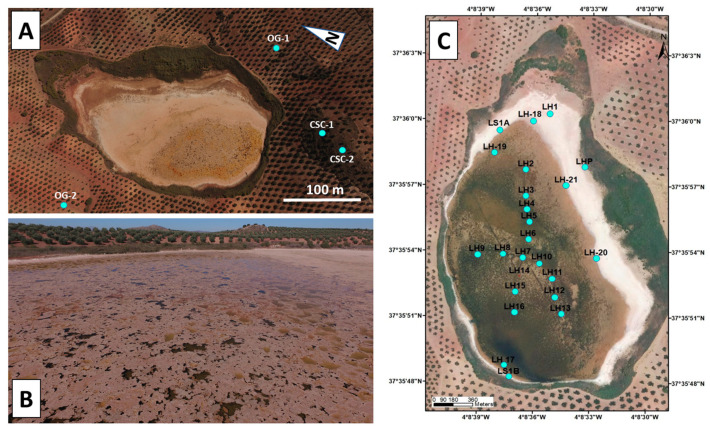
(**A**) Flooded surface area of Laguna Honda surrounded by olive groves. Location of the sampling points where samples of soils from the surrounding olive groves (OG) and of sediments of the chaotic subbetic complexes (CSC) were taken are indicated in the figure. (**B**) Seasonal pale pink floating algal mats covering the water surface of Laguna Honda during spring and summer. (**C**) Location of the sampling points where cores of sediments deposited on the bottom of the lake up to 30 cm deep were taken.

Laguna Honda wetland is located in one of the chaotic subbetic complexes (CSCs) included in the post-orogenic Guadalquivir River basin at the point of contact with the External Zone of the Betic Cordillera (Figure 1A,B). CSCs include evaporitic Triassic, Cretaceous, and Miocene olistoliths from the External Betic Zone resedimented in the middle Miocene gypsum-rich unit of the Guadalquivir River Basin [20,21,22]. The geological substrate and materials surrounding Laguna Honda wetland are marls, clays, gypsum, and isolated blocks of carbonate rocks (limestone and dolostone) (Figure 1C). A karstic morphogenetic system formed by the dissolution of the evaporitic materials of the area and fed by groundwaters and surface waters gave rise to the Laguna Honda wetland. The floodable area of the wetland is around 8.5 ha, and the catchment area occupies 96.2 ha. The area surrounding the wetland is completely dedicated to the monoculture of olives (Figure 2A). The olive groves are planted on high-slope areas, especially on the southern shores of the wetland lake. Tillage operations in the olive groves comprise the main soil management processes, which permanently maintain the bare soil. This inadequate agricultural practice promotes intense soil degradation processes.

Regarding the bathymetry of the wetland lake, the depth progressively increases from the NE to the SW. A deltaic zone has developed on the northern shores of the lake. This area frequently emerges during drought periods. The deepest area is located in the permanently flooded southern part of the lake, where a depth of 2.5 m is reached.

The sediments of the wetland are colonized by submerged macrophytes, charophytes, and helophytes (*Ruppia drepanensis*, *Chara galioides*, *Najas marina*) [24]. The use of fertilizers in the surrounding olive groves promotes the eutrophication of waters, producing seasonal algal blooms (Figure 2B).

The climate of the area is characterized by semi-arid conditions very close to a subhumid environment, with an average temperature of 12 °C during winter and 30 °C during summer. The average annual rainfall is 551 mm, although this is frequently concentrated in high-intensity rainfall episodes occurring during the wet season (October to May), which alternates with a prolonged dry season [25].

An extended characterization of the wetland origin and hydrochemistry, vegetation composition, mineral sediment constituents, and inhabitant microorganisms can be found in Medina et al. [26,27,28,29]. The wetland waters are hypersaline (salinity around 48 PSU) and alkaline (pH > 9.2) (Table 1). These waters show very elevated electrical conductivity (EC) (up to 73 mS/cm), positive oxidation–reduction potential (ORP) values in the surface waters, and a negative ORP near the bottom of the lake (up to −215). The anionic composition is characterized by very elevated SO_4_^2−^ (>24,000 mg/L) and Cl^−^ (>21,000 mg/L) contents and significantly high cation concentrations of Na^+^ (up to 11,600 mg/L), Mg^2+^, and Ca^2+^ (up to 8700 mg/L and around 1000 mg/L, respectively).

The sediments of the lake are characterized by an upper black silty-clayey layer rich in organic matter (TOC average around 1.44%) and a lower bed rich in clays (TOC average around 0.7%) (Table 2). The sediment pH is lower in the shore zones (up to 6.33) than in the central part of the lake (up to 8.54). The sediment Eh values oscillate between −80.9 mV in the central deep part of the wetland and an average of −13.6 mV in the shore part. High electrical conductivity (up to 17.36 mS/cm) and salinity (2.49 g/L) are also observed in the sediments from the shores.

**Figure 3:**
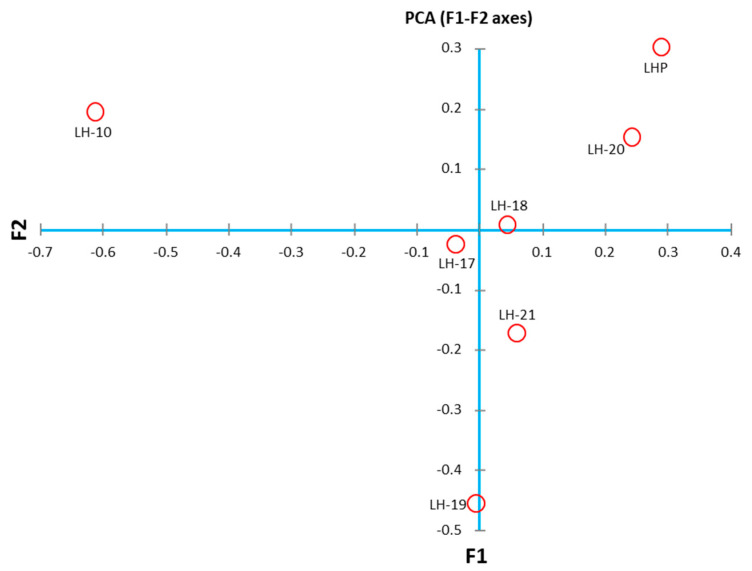
Principal coordinates analysis (PCA) of samples for the determination of the bacterial diversity in the sediments from Laguna Honda (modified from Medina et al., [29]).

The microbiological data from Medina et al. [27,28,29] revealed that the sediments from Laguna Honda have a broad microbial diversity. Proteobacteria is the best-represented phylum, with *Thioalkalispira-Sulfurivermis*, *Thiohalophilus*, *Candidatus* Thiobios (and other members of the *Chromatiaceae* family), and *Thiobacillus* genera in the shore sediments (up to 24.89) and *Salinivibrio* and *Vibrio* in the sediments from the central part of the wetland (up to 10%) (Table 3, Figure 3). *Plantomycetota* communities are important in deep sediments with Fam. AKAU_sediment_group (17.98%) but are less relevant in shore zones with Fam. *Gimesiaceae* (4.13%). The phylum *Desulfobacterota* is present in all the sediments of the lake (up to 14.28%), with Fam. *Desulfosarcinaceae*, *Desulfatiglandaceae*, *Desulfobacteraceae*, *Desulfobulbaceae*, and *Desulfurivibrionaceae*. The *Chloroflexi* phylum is important in sediments richer in organic matter, with members including *Anaerolineae* and *Dehalococcoidia*. Emerged sediments are characterized by a significant presence of the genus *Sulfurovum* from the *Campilobacterota* phylum (8.95%). Some sediments have significant communities of *Acidobacteriota* (*Aminicenantales*), *Spirochaetota* (*Spirochaeta_2*), *Verrucomicrobiota* (*Candidatus* Omnitrophus, DEV007 and *Luteolibacter*), *Nitrospirota* (*Thermodesulfovibriona*), and *Zixibacteria*.

The mineralogical composition of the sediments is characterized by high contents of silicates. Illite and chlorite are the main clay minerals in the sediments (around 45%) and define a microlamination that includes grains of quartz, feldspars, organic fragments, aggregates of carbonates (aragonite, calcite, and dolomite), gypsum, and pyrite framboids. Halite, celestine, and anhydrite can be present in minor amounts in some samples.

### 2.2. Methods

The experimental procedures employed to obtain the physicochemical parameters of the waters and sediments, including X-ray diffraction, as well as the TOC analysis and microbiological characterization indicated in the previous section, are described in Medina et al. [29], and samples were obtained from 24 sampling points (Figure 2C). A network made from two transversal sections was created to select the sampling points: an approximately N–S main section parallel to the elongation of the wetland lake and another approximately E–W section. The sampling points were separated by approximately 150 m in the network. The characterization of the sediments allowed us to distinguish two main groups of materials: sediments from the shore and the central part of the lake. Given the homogeneity of the sediments in each group, nine representative and regularly spaced shore and central sampling points were selected to characterize the presence of gold in the sediments from the Laguna Honda wetland. The presence of gold grains in sediments from these sampling points was assessed using field emission high-resolution scanning electron microscopy (FESEM) and high-resolution transmission electron microscopy (HRTEM). Once checked for the presence of gold particles, the samples were selected for the bulk Au content analysis of the sediment. The selected sampling points for gold analysis are indicated in Table 4. The cores of sediments deposited on the bottom of the lake were taken from a depth of 0 to 30 cm. The cores were obtained using PVC tubes sealed with plugs and stored in a portable cooler at temperatures below 5 °C. The upper part of the cores consisted of dark silty-clay sediments, whereas further down, the cores contained brownish and clay-silty sediments. According to these features, three samples were extracted from each core for the geochemical analysis of gold: an upper segment (up to 3 cm) with dark silty-clay sediments (labelled with number 5 in Table 4), a middle segment of sediments (from around 11 to 14 cm; labelled with number 3 in Table 4), and a deeper segment (from around 27 to 32 cm) with brownish clay-silty sediments (labelled with number 1 in Table 4). Moreover, in order to provide information on the regional background gold concentration, two samples of soils from the olive grove surrounding the wetland and two marly sediments from the CSCs, where Laguna Honda formed, were also selected for geochemical analysis, which included a total of 31 samples.

A Carl Zeiss MERLIN high-resolution scanning electron microscope (FESEM) with EDX analytical capability (SIC, University of Jaén, Spain) was used to obtain analytical and textural data through secondary electron (SE) imaging (see “[30]” for details). For this purpose, sediment samples were mounted on supports and metallized with carbon. In addition, a backscattered electron (BSE) detector was used to complete the characterization of minerals with metallic content.

The nanometric characterization was carried out by means of high-resolution transmission electron microscopy (HRTEM) following the experimental procedure indicated by Nieto et al. [31]. For the compositional characterization of the clays, whose grain size is often outside the resolution of the SEM, transmission electron microscopes (HRTEMs), namely the HAADF FEI TITAN G2 microscope, operating at 300 kV, and the HRTEM HAADF Thermo Fisher TALOS, operating at 200 kV, were used (Scientific Instrumentation Centre, University of Granada). Chemical analyses of the nanoparticles were performed on powders dispersed over gold or nickel grids in scanning transmission electron microscope mode with an energy-dispersive X-ray spectroscopy (AEM-EDX) microanalysis system.

Samples of sediments for gold analyses were dried at 105 °C for 48 h, ground, and acid-digested using HNO_3_:H_2_O_2_:HCl (6:3:2). After dilution, the total Au concentration was measured using a Thermo Finnigan-High Resolution ICP-MS by ACTLAB Laboratories (Ancaster, ON, Canada) and an ICP-MS with a NexION 300D (CIC, University of Granada). Certified standards were used for quantification (BR-N, GH, DR-N, UB-N, AGV-N, MAG-1, GS-N, and GA). The detection limit was below 1 μg/kg. The absence of drift during the Au analysis was verified by measuring two Au standards (5 and 10 ppb, Cole-Parmer) every ten samples. The standard deviation was always less than 5%.

Microbial DNA was extracted with a DNeasy PowerSoil Kit (Quiagen, Hilden, Germany) and quantified with a QuantiFluor^®^ ONE dsDNA system (Promega, Madison, WI, USA). For the metagenomic sequencing, library preparation of the 16S rDNA gene amplicons was performed following the Illumina protocol (Cod. 15044223 Rev. A) targeting the 16S rDNA gene’s V3 and V4 region. The primers were selected from Klindworth et al. [32]. The following 16S rDNA gene amplicon PCR primer sequences were used [32]: forward primer, 5′TCGTCGGCAGCGTCAGATGTGTATAAGAGACAGCCTACGGGNGGCWGCAG3′; reverse primer, 5′GTCTCGTGGGCTCGGAGATGTGTATAAGAGACAGGACTACHVGGGTATCTAATCC3′. Illumina adapter overhang nucleotide sequences were added to the gene-specific sequences. The protocol for 16S rDNA amplification was initiated with microbial genomic DNA (5 ng/μL in 10 mM Tris pH 8.5). The multiplexing step was performed using a Nextera XT Index Kit (FC-131-1096). After a quality check, the libraries were sequenced using a 2 × 300 pb paired-end run (MiSeq Reagent kit v3 (MS-102-3001)) on a MiSeq Sequencer (Illumina, San Diego, CA, USA). The sequence data were checked with the prinseq-lite program [33]. The DADA2 pipeline [34] was used for denoising, paired-end joining, and chimera depletion steps. Sequences were then analyzed using the qiime2 pipeline [35]. Taxonomic affiliations were assigned using the naive Bayesian classifier integrated with the qiime2 plugins and the SILVA_release_132 database [36]. Statistical analysis was carried out with SPSS software version 24 (IBM Corp., Foster City, CA, USA).

## 3. Results

### 3.1. Regional Background and Gold Concentration in Sediments

The soil samples surrounding the wetland and the sediments from the CSCs where Laguna Honda formed have Au contents below the detection limit (Table 4).

However, the wetland sediments were enriched in gold. The gold concentrations in the sediments from the Laguna Honda wetland varied according to the sediment location and depth (Table 4). Au was enriched in the sediments from the northern shore. The highest Au contents are found in the upper layer of these sediments (21.90 ppm). The Upper layers of sediments of the central part of the lake have lower Au contents (1.89–0.72 ppm) than the upper layers from the northern shores (up to 2.31 ppm). The Au concentration decreases with sediment depth, with the lowest values varying from 0.63 ppm in the northern sediments to 0.19 ppm in the southern part of the lake.

### 3.2. FESEM and HRTEM Characterization

**Figure 4:**
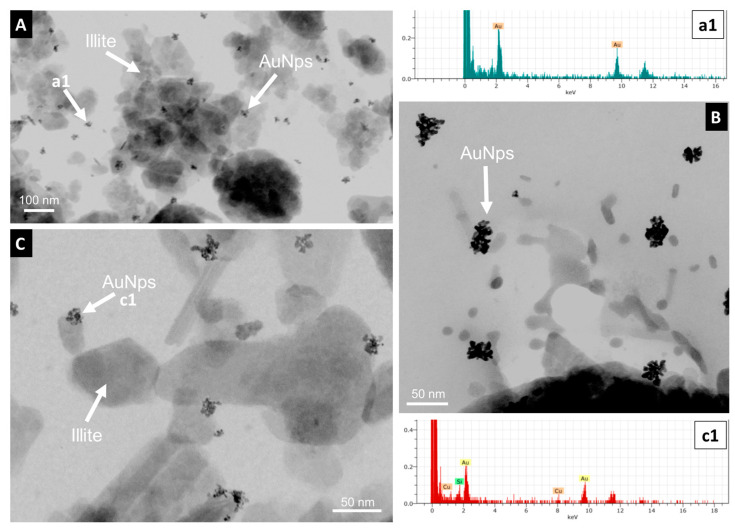
HRTEM images and EDX analyses of the Au-rich sediments. (**A**–**C**) Small dispersed groups (nanoaggregates around 10 nm) made from several AuNp (arrows) sometimes adhering to illite (right arrow), in this case forming heteroaggregates of AuNp-mica. Spectra (**a1**,**c1**) EDX analyses of the particles shown in (**A**,**C**).

**Figure 5:**
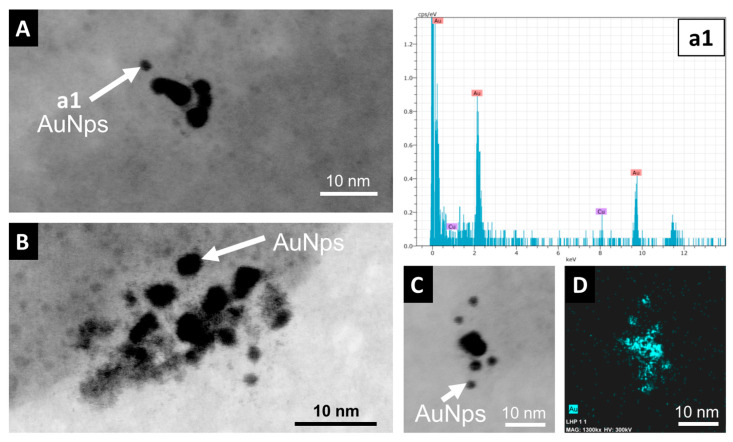
Details of very small Au nanoparticle groups in HRTEM images and EDX data. (**A**–**C**) HRTEM images showing details of small-sized irregularly shaped gold nanoparticles. Spectrum (**a1**) EDX analysis of the particle indicated in (**A**). (**D**) Elemental map (HRTEM-EDX) of Au (image (**C**)).

The nano- to micrometer characterization of AuNp distribution was performed using scanning and transmission electron microscopy. Transmission electron microscope images of deep sediments from the central part of the wetland, the shore, and emerged sediments revealed the presence of small dispersed nanoaggregated gold nanoparticles between 10 and 40 nm in size (Figure 4) that exhibit a skeletal texture (Figure 4B). A few isolated non-aggregated nanoparticles clearly isolated with a diameter of around 2 to 4 nm can be observed (Figure 5), making it difficult to establish the original size of the emitted nanoparticles.

**Figure 6:**
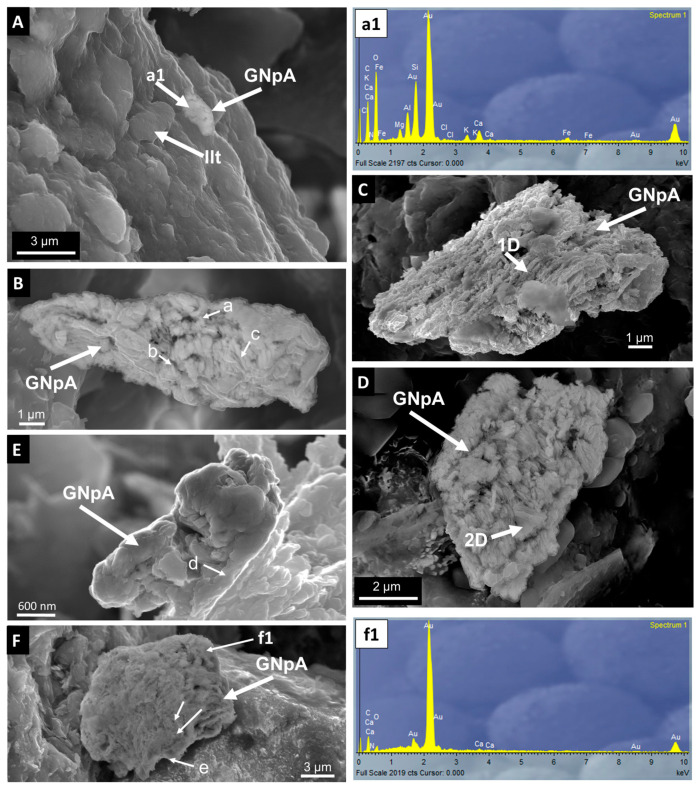
Secondary electron images of assembled gold nanoparticle aggregates (GnpA). (**A**) Heteroaggregate AuNp-mica intercalated between the lamination defined by the deposition of the illite grains. Spectrum (**a1**) EDX analysis of the GnpA indicated in (**A**). (**B**) Homoaggregates of fused AuNp interconnected by thin irregular random filaments of Au (a), thicker elongated particles around 50 nm wide and up to 1 µm long (b) or showing many 1D aggregates with very close contacts or even fused together (c), forming a characteristic inner porous mosaic texture leaving irregular voids. (**C**) Gold nanoparticle homoaggregate where 1D structures are predominant. (**D**) Gold nanoparticle homoaggregate where 2D structures can be observed. (**E**) Details of a AuNp homoaggregate with an accumulation of 1D aggregates covered by a thin layer of two-dimensional aggregates made from fused AuNp (d). (**F**) AuNp showing dissolution marks (arrows) and covered by a thin layer of fused AuNP. Spectrum (**f1**) EDX analysis of the GNpA indicated in (**F**).

Most nanoparticles of the dispersed nanoaggregates are fused together, but some others are separated by a space of <1 nm (Figure 5). Figure 4A,C show that the small nanoparticle groups frequently adhere to platy pseudohexagonal crystals of detrital illite (mica) to form heteroaggregates of AuNp-mica. These heteroaggregates are frequently intercalated between the lamination defined by the deposition of the illite grains (Figure 6A).

Shore and emerged sediments were characterized by the presence of large three-dimensional (3D) structures (up to 20 µm) of assembled gold nanoparticle homoaggregates (Figure 6B). The inner part of the 3D structures was made from interconnected one-dimensional (1D) aggregates of fused gold nanoparticles.

One-dimensional aggregates appear as very thin irregular random filaments (a in Figure 6B) or thicker elongated particles around 50 nm wide and up to 1 µm long (b in Figure 6B). Many 1D aggregates had very close contacts or were even fused together (c in Figure 6A), and they are parallelly arranged forming a characteristic inner porous mosaic texture, leaving irregular voids (Figure 6B–F). One-dimensional structures were predominant in most of the gold nanoparticle homoaggregates (e.g., Figure 6B,C), although 2D structures can also be observed in many gold nanoparticle homoaggregates (e.g., Figure 6D). The accumulation of 1D and 2D aggregates was commonly covered by a thin layer of two-dimensional aggregates made from fused gold nanoparticles (d in Figure 6E, e in Figure 6F).

## 4. Discussion

The fate of gold nanoparticles emitted into aquatic systems is the result of different types of environmental interactions, determining their permanence as suspended particles, their aggregation and deposition in sediments, and their solubility and bioavailability. Physicochemical conditions (temperature, hydrodynamics, Eh, pH, salinity, the presence of reactive ions, etc.) and the occurrence of organic and inorganic particles and nanoparticles are some of the main factors controlling the environmental transformation processes of the emitted gold nanoparticles [12,13,37]. Moreover, possible AuNp biotransformations in systems with high biological activity must be taken into account, in spite of the supposed strong environmental stability of these particles, e.g., [7].

### 4.1. Origin of the Gold Concentration: Geological Background

From the geological point of view, the Laguna Honda wetland was formed by karstic processes on the sedimentary materials of the chaotic subbetic complexes (CSCs) at the point of contact between the Guadalquivir basin and the External Zone of the Betic Cordillera (Figure 1A,B). Hydrothermal gold deposits or regional placer gold mining have not been described in the available geological information on this region.

Wetland sediments are enriched in gold as regards the Au content of the geological materials in the study area. The chemical analyses of the geological substrate and the soils around the wetland (Table 4) reveal that the background gold concentration of the area is very low. The soil samples surrounding the wetland and the sediments from the CSCs where Laguna Honda formed have Au contents below the detection limit, indicating that the concentration of gold in the wetland sediments is not related to the regional background, suggesting a pollution process.

### 4.2. Aggregation Processes

Particle deposition is favored when aggregation processes producing large and heavy accumulates occur in low-energy aquatic systems. The importance of aggregation processes has previously been highlighted in experimental studies [11,38,39,40]. These studies predict nanoparticle fate in aquatic environments, mimicking ecosystem complexity in experimental micro- or mesocosmos using physical and chemical determinations of the environmental nanoparticle permanence. However, the fate of the nanoparticles is not frequently documented in images obtained by means of high-resolution electron microscopy techniques. The lack of this type of information is more evident in the accumulation of anthropogenic gold nanoparticles in natural aquatic environments; in spite of this, electron microscopy images allow us to directly observe the geochemical processes affecting their behavior. In this work, electron microscopy reveals morphological differences between the aggregates observed in the sediments from the shore and those from the central part of the Laguna Honda wetland lake, suggesting different types of interactions between gold nanoparticles and detrital sedimentary particles transported to the lake.

#### 4.2.1. Rapid Deposition of Large Au Homoaggregates in the Shore Wetland

This study reports SEM images of large AuNp homoaggregates up to 20 µm in size in the shore and emerged sediments of the lake, where the Au concentrations reach the highest concentrations (up to 21.9 ppm). The homoaggregates of gold nanoparticles observed in the Laguna Honda wetland do not show the same morphologies as those observed in natural Au particles collected in fluvial and lacustrine environments, which are characterized by a bigger size (up to 2 mm), frequently being rounded with the presence of signs of physical transformations occurring during and post-transport. Folded edges, striations, randomly orientated scratches, and pitted cavities are commonly indicative of alluvial transport [41]. With increasing fluvial transport, particles acquire battered and roughened or rounded surface morphologies [41,42,43]. The lack of these morphological features in the Laguna Honda homoaggregates suggests a short transport process.

Surette and Nason [9] showed that homoaggregation is dominant relative to heteroaggregation for most types of gold nanoparticles in river waters. Avellán et al. [7] indicated that the addition of high concentrations of metallic nanoparticles in experimental mesocosmos produces homoaggregation processes. According to the observations of Geitner et al. [44], Avellan et al. [7] suggested that gold nanoparticles should have a similar behavior to other metallic nanoparticles resistant to dissolution, tending to accumulate in stable forms in the sediment compartment of the environment. Yecheskel et al. [45] indicated that the presence of divalent cations (Ca^2+^ and Mg^2+^) can enhance AuNp aggregation in the soil columns. Therefore, the presence of large AuNp homoaggregates in the shore and emerged sediments from the Laguna Honda wetland suggests a high collision frequency of gold nanoparticles in the Mg- and Ca-rich waters feeding Laguna Honda lake due to the high particle number and concentration, likely facilitated by the inappropriate application of pesticides, promoting the rapid coagulation of heavy and compact homoaggregates that were quickly deposited in the shores of the lake. Sponge-like Au aggregates formed by chain-like fused structures of AuNPs similar to those observed in the shore sediments from Laguna Honda have been described in experiments at high concentrations of added Au nanoparticles [46,47,48], especially in low-pH conditions where aggregated Au chains can be easily condensed from the Au colloid.

#### 4.2.2. Transport of Au-Illite Heteroaggregates to the Central Part of the Wetland

On the other hand, attempts to obtain SEM images of gold aggregates in the permanently flooded sediments from the central part of the lake characterized by lower but still significant Au concentrations (up to 1.89 ppm) were unsuccessful. Gold was only detected in these sediments as scattered particles in the TEM images, frequently adhering to sedimentary illite grains to form small heteroaggregates. Several studies [44,45,46] have revealed that high-pH environments with high availability of illite favor sorption processes, mainly driven by the electrostatic attraction between negatively charged AuNPs and positively charged illite edges. Fu et al. [46] provided TEM observations where AuNPs were mainly attached to illite edges, as similarly occurs in the illite-rich sediments from Laguna Honda.

Therefore, the presence of illite particles suspended in the waters of Laguna Honda coming from the weathering and transport of the sedimentary surrounding materials can affect the aggregation, transport, and deposition processes of the gold nanoparticles. Medina et al. [29] revealed that the sediments deposited in Laguna Honda are mainly made from silicates, with illite being the most abundant clay mineral (up to 60%), as well as some authigenic carbonates and sulfates. Sastre et al. [49] indicated that erosion in Mediterranean olive groves is reinforced by hilly terrain, bare soil, and high-intensity rainfall. The abundant influx of illite into the Laguna Honda lake was produced by the natural weathering, erosion, and transport of the CSCs surrounding reliefs favored by negative agricultural practices, e.g., [50,51], leading to the absence of vegetation cover in the olive groves surrounding the wetland [29].

The experimental study of Thio et al. [52] showed that aquatic systems with organic matter and high ionic strength and salinity favor the accumulation of gold nanoparticles on the mica surface, whereas, at the same ionic strength, alkaline environments are less favorable for the adhesion of gold nanoparticles. Gallego-Urrea et al. [11] suggested that the presence of illite particles in aquatic systems can produce a differential flocculation process affecting the deposition of gold nanoparticles. Thus, when gold nanoparticles are heteroaggregated with illite particles, low-density flocs are formed, which settle more slowly than the higher-density homoaggregates of gold nanoparticles. The interaction of a significant amount of gold nanoparticles emitted due to the inappropriate application of pesticides with the high influx of illite particles into the hypersaline waters of Laguna Honda facilitated the simultaneous formation of Au-illite heteroaggregates with a lower density than the Au homoaggregates, which are quickly formed and deposited in the shore sediments. Au-illite heteroaggregates were transported into the central part of the lake where the accumulation of additional AuNP increased their density and favored their deposition as scattered particles in the central sediments.

### 4.3. Dissolution Hazard

Solubilization of AuNps is one of the most important processes that facilitate the entrance of Au into the biogeochemical cycle, with implications for health, safety, and environmental toxicity [13,37]. Monitoring the presence of textural evidence of AuNp dissolution is important to reveal AuNp transformations potentially affecting the bioavailability of these metallic nanoparticles in aquatic environments.

The TEM images reveal an irregular morphology of gold nanoparticles, with a size smaller than that of gold nanoparticles commonly used in pesticides, while they are also frequently fused. Fused nanoparticles are also reported in the SEM images of the large aggregates formed in the shore sediments from the Laguna Honda wetland. Pattadar et al. [53] observed that aggregates with strongly fused contacts between AuNp increase their oxidation potential, which could indicate that fused contacts can be the result of dissolution–precipitation processes associated with the oxidative mobilization of gold. Malejko et al. [5] showed a gradual decrease in the gold nanoparticle size produced by the partial dissolution of nanoparticles used in soils of different crops, which can be followed by a stage of ionic uptake and reformation of nanoparticles and aggregates. The small size of the AuNp observed in HRTEM (10 times smaller than those frequently used in agricultural products, Figure 5) and the presence of fused contacts in the nano- and micro-organization of the aggregates evidenced by SEM and HRTEM images (Figure 4, Figure 5 and Figure 6) suggest that the stability of the AuNp was affected by dissolution processes that modified their original inert properties and affected their behavior. The presence of similar small elemental Au nanoparticles to those observed in the Laguna Honda sediments has been described as a result of gold mobilization processes commonly associated with Au geomicrobiology [54].

Most of the models predicting the fate of gold nanoparticles in natural environments are based on the behavior of experimental systems with low biotic complexity exclusively considering the effect of abiotic transformations, which can lead to misinterpretations about the reactivity of gold particles and their toxicity. However, there is strong evidence that biologically mediated transformations can occur in environments with high biological activity [7,12]. Solubilization via the microbe-mediated oxidation of Au^0^ and later complexation with different types of ions emitted by microorganisms (e.g., thiosulfate, cyanide) has been proposed in several studies [55,56,57,58]. Sulfur-oxidizing bacteria (SOB) and sulfate-reducing bacteria (SRB) are important microorganism communities populating hypersaline wetlands, determining the sulfur cycle, and playing important roles in the fixation or release of metals, e.g., [30,59]. Dhiman et al. [60] revealed that important communities of SOB exhibit features that promote plant growth, such as the production of hydrogen cyanide, the presence of which minimally affects its microbial activity [61], although some other SOB groups, such as *Thiobacillus*, are used to remove cyanide from polluted systems. The presence of cyanide in the environment coming from microorganism activity is one of the most important chemical factors promoting the oxidation and complexation of gold [7,56].

In sulfate- and organic matter-rich systems, such as hypersaline wetland environments, the decay of organic matter is mostly related to biologically mediated processes of sulfate reduction, where SRB plays a main role [62,63]. The reduction process culminates with the generation of sulfides, but other stable sulfur phases, which are intermediate in the natural sulfur cycle, such as thiosulfates, are produced. The oxidation of authigenic sulfides mediated by SOB can also produce thiosulfates [64]. Biogenic thiosulfates are frequently used as leaching lixiviants in carbon-rich gold ores due to their ability to oxidize and complex Au^0^ [52,65,66,67].

Medina et al. [29] revealed the presence of relevant populations of SRB and SOB in the Laguna Honda sediments. SRB from the *Desulfobacterota* (*Desulfatiglandaceae* fam. predominant in deep sediments and *Desulfobacteraceae*, *Desulfosarcinaceae*, *Desulfobulbaceae*, and *Desulfurivibrionaceae* predominant in shallower sediments and emerged areas) and *Nitrospirota* phyla (C. *Thermodesulfovibrionia* in shallow sediments) were found in all of the wetland compartments. The metabolism of *Desulfobacterota* members produces thiosulfate as a sulfur cycle intermediate, e.g., [68], whereas *Thermodesulfovibrionia* communities are involved in the disproportionation of thiosulfates. SOB from *Campylobacteria* (Genus *Sulfurovum*) and *Gammaproteobacteria* (genera *Thiobacillus*, *Thioalkalispira-Sulfurivermis*, *Candidatus Thiobios*, and other S purple bacteria of the *Chromatiaceae* family) were abundant in the shore areas of Laguna Honda. Yates et al. [69] revealed that in some microbial electrosynthesis processes, *Chromatiaceae* microbial communities grow on gold cathodes and can reduce O_2_ using gold as an electron donor. Therefore, the metabolism of SRB and SOB in the Laguna Honda wetland could provide the environment with different compounds (such as thiosulfate or cyanide) involved in the dissolution and transport of gold and other metals as occurred in the studies of Avellan et al. [12] and McGivney et al. [13]. The presence of very small irregularly shaped gold nanoparticles (Figure 5) and textural evidence of fused particles that can be associated with dissolution in the gold aggregates (Figure 4, Figure 5 and Figure 6) suggest that the gold nanoparticles emitted by nanopesticide treatments of olive groves surrounding the Laguna Honda wetland experienced processes of dissolution. Shore sediments inhabited by an important SOB community and characterized by a high Eh and low pH, likely caused by the microbial production of acid compounds, were especially favorable for the dissolution of scattered gold nanoparticles and heavy homoaggregates. Moreover, the shore sediments almost always contained bacterial communities resistant to heavy metal stress (*Luteolibacter* and *Maricaulis*). *Luteolibacter* has been considered to be a hydrolytic bacteria involved in the dissolution of metals and the decay of polycyclic aromatic hydrocarbon [70,71,72]. Maricaulis possesses membrane-bound proteins able to transport metals [73,74].

## 5. Conclusions

1. The high gold content of the hypersaline Laguna Honda shore sediments resulted from the formation of heavy AuNp homoaggregates. These aggregates were formed by the high-frequency collision of nanoparticles promoted by the inappropriate application of a high concentration of pesticides.
2. Lighter AuNp-illite heteroaggregates were formed by the interaction of AuNp with a high influx of illite into the lake. The illite input was produced by reinforced erosion processes of the surrounding olive groves produced by negative soil management.
3. AuNp shows micro and nanotextural evidence of instability, such as the very small size of the isolated nanoparticles and fused contacts of particles in the aggregates.
4. The metabolic activity of the important microbial communities of SRB and particularly SOB in the shore sediments displays the ability to provide compounds (such as thiosulfate or cyanide) that decrease the pH and increase the Eh of the sediments. These conditions promote AuNp modification by means of the dissolution process and the transport of gold. Bacterial communities resistant to heavy metal stress (such as *Luteolibacter* and *Maricaulis*) can also contribute to AuNp destabilization and transport.

## Figures and Tables

**Table 1:** Summary of the physicochemical properties of the Laguna Honda column waters. PSU: Practical Salinity Unit, ORP: oxidation and reduction potential (data from Medina et al., [29]).

		Mean	Min.	Max.
Conductivity	mS/cm	71.62	70.88	73.00
Salinity	PSU	47.56	46.97	48.20
pH		9.40	9.20	9.55
Eh	mV	−132.54	−165.00	−111.00
ORP		−0.30	−215.00	150.50
HCO_3_^−^	mg/L	24.72	17.76	40.63
CO_3_^2−^	mg/L	67.20	58.01	70.42
Cl^−^	mg/L	21,633.12	21,370.29	22,203.93
NO_2_^−^	mg/L	2.52	0.00	12.59
Br^−^	mg/L	63.53	29.34	72.87
NO_3_^−^	mg/L	48.06	46.84	50.38
SO_4_^2−^	mg/L	24,537.05	24,142.58	24,840.72
Na^+^	mg/L	11,461.16	11,348.04	11,601.27
K^+^	mg/L	144.60	143.10	147.84
Ca^2+^	mg/L	1058.25	1016.64	1082.41
Mg^2+^	mg/L	8566.24	8489.00	8667.02
Sr^2+^	mg/L	22.52	21.07	24.78

Max: maximum; Min: minimum.

**Table 2:** Summary of the physicochemical properties of the Laguna Honda sediments. TOC: total organic carbon (data from Medina et al., [29]).

		Mean	Min.	Max.
TOC	mS/cm	1.03	0.17	2.25
Salinity	PSU	1.83	1.56	2.49
pH		7.54	6.33	8.54
Eh	mV	−26.26	−80.90	26.40
Conductivity	mS/cm	13.08	10.48	17.36
Chlorite	%	5	1	21
Illite	%	40	5	60
Quartz	%	21	7	45
Calcite	%	15	4	37
Gypsum	%	12	0	72
Dolomite	%	3	0	8
Aragonite	%	2	0	19
Halite	%	1	0	4
Pyrite	%	1	0	13

Max: maximum; Min: minimum.

**Table 3:** Relative abundances (%) of sediment microbial communities from Laguna Honda. Location of samples in Figure 2C (data from Medina et al., [29]).

Taxonomic Group/Sample	1	2	3	4	5	6	7
*o__Methanofastidiosales (uncultured)*	2.41	0.00	0.00	0.00	0.00	0.00	0.00
*o__Methanomassiliicoccales*	0.03	1.48	1.12	2.42	1.87	0.38	0.57
*g__Acetothermiia*	2.39	0.01	2.42	0.20	0.02	0.16	0.01
*g__Aminicenantales*	0.38	7.93	1.23	0.48	2.26	0.61	0.13
*g__Sulfurovum*	0.00	0.00	0.18	0.19	0.18	0.06	8.95
*f__Anaerolineaceae (uncultured)*	0.54	2.44	2.21	1.23	2.41	2.12	1.35
*g__SBR1031*	2.08	2.48	0.60	0.34	0.63	1.15	0.46
*g__GIF3*	0.33	2.30	0.24	0.05	0.21	0.25	0.00
*g__MSBL5*	2.80	0.93	4.79	0.19	0.36	0.83	0.01
*g__Babeliales*	0.06	0.29	2.45	0.29	0.22	0.32	0.19
*g__Desulfatiglans*	1.39	4.13	1.93	1.14	1.83	2.23	0.90
*g__Desulfotignum*	0.00	0.00	1.21	5.74	0.00	0.71	0.34
*f__Desulfosarcinaceae (Others)*	4.06	1.96	5.67	1.52	4.81	2.74	2.70
*f__Desulfosarcinaceae (uncultured)*	0.11	0.09	2.59	0.48	0.50	0.63	0.22
*f__Desulfobulbaceae (Others)*	0.19	0.61	0.81	0.22	3.12	0.69	2.58
*g__MSBL7*	0.98	0.17	0.05	0.00	0.07	2.16	1.38
*g__BD2-11_terrestrial_group*	0.80	2.57	0.69	1.49	2.59	2.59	2.02
*c__Thermodesulfovibrionia*	0.00	1.71	1.41	1.43	3.76	1.01	0.58
*g__AKAU3564_sediment_group*	17.98	1.24	0.23	0.28	0.12	0.26	0.09
*f__Gimesiaceae (uncultured)*	0.00	0.00	0.13	0.00	0.30	0.07	3.93
*g__Maricaulis*	0.05	0.00	0.00	0.00	0.10	0.06	3.16
*g__Magnetovibrio*	0.03	0.41	0.42	0.00	3.14	2.09	0.51
*c__Gammaproteobacteria (Others)*	0.16	1.85	0.43	2.53	0.80	4.27	0.37
*g__Thiobacillus*	0.00	0.03	3.51	0.31	5.47	0.47	6.80
*g__Candidatus_Thiobios*	0.00	0.00	0.19	3.55	0.03	0.26	0.01
*f__Chromatiaceae (uncultured)*	0.00	0.00	0.74	0.07	8.85	0.10	0.20
*g__Thioalkalispira-Sulfurivermis*	0.00	4.08	2.47	0.58	4.75	2.90	4.95
*g__Thiohalophilus*	0.03	0.00	0.06	0.00	0.16	5.25	0.21
*o__Gammaproteobacteria_Incertae_Sedis (uncultured)*	0.02	0.12	0.36	0.21	1.32	0.25	2.74
*g__Salinivibrio*	3.89	0.00	0.00	0.00	0.00	0.00	0.00
*g__Vibrio*	6.09	0.00	0.00	0.02	0.00	0.00	0.00
*g__Spirochaeta_2*	1.17	0.17	0.84	3.01	1.26	0.96	0.38
*c__Sumerlaeia (uncultured)*	0.71	7.87	2.18	0.51	1.15	0.66	0.67
*g__Candidatus_Omnitrophus*	0.92	2.59	2.08	1.19	0.90	2.20	0.22
*g__DEV007*	0.00	0.00	0.09	2.68	0.00	0.07	0.02
*g__Luteolibacter*	0.05	0.00	0.28	0.31	4.61	0.00	0.33
*g__Zixibacteria*	1.80	0.00	0.82	0.40	0.12	2.11	2.00
*Rest (<2.0%)*	48.55	52.55	55.59	66.98	42.06	59.38	51.03

Samples: 1: LH-10, 2: LH-17, 3: LH-18, 4: LH-1, 5: LH-20, 6: LH-21, 7: LHP.

**Table 4:** Gold concentrations in sediments from the Laguna Honda wetland.

Sampling Point	Depth	Au ^1^
LHP,5	0–2 cm	21.90
LHP,3	11–13 cm	2.41
LHP,1	30–32 cm	0.75
LH-1,5	2–3 cm	2.31
LH-1,3	13–14 cm	1.43
LH-1,1	27–28 cm	0.63
LH-3,5	2–3 cm	1.89
LH-3,3	13–14 cm	1.00
LH-3,1	27–28 cm	0.37
LH-5,5	2–3 cm	1.65
LH-5,3	13–14 cm	1.02
LH-5,1	27–28 cm	0.43
LH-7,5	2–3 cm	0.97
LH-7,3	13–14 cm	0.75
LH-7,1	27–28 cm	0.32
LH-9,5	2–3 cm	0.72
LH-9,3	13–14 cm	0.68
LH-9,1	27–28 cm	0.23
LH-11,5	2–3 cm	0.79
LH-11,3	13–14 cm	0.81
LH-11,1	27–28 cm	0.25
LH-13,5	2–3 cm	0.72
LH-13,3	13–14 cm	0.69
LH-13,1	27–28 cm	0.19
LH-15,5	2–3 cm	1.45
LH-15,3	13–14 cm	1.12
LH-15,1	27–28 cm	0.43
OG-1	-	<0.001
OG-2	-	<0.001
CSC-1	-	<0.001
CSC-2	-	<0.001

^1^ ppm. LHP and LH: Wetland sediments. OG: Olive grove soils. CSC: Substrate sediments. Locations indicated in Figure 2.

## Data Availability

Data are contained within the article.
